# The Effect of Trapidil and Bevacizumab on Tracheal Anastomotic Wound Healing^☆^

**DOI:** 10.1016/j.curtheres.2013.04.002

**Published:** 2013-12

**Authors:** Esra Ertan, Perihan Göçer, Ali Naycı, Ülkü Çömelekoğlu, Sibel Atış, Rabia Bozdağan Arpacı, Gülden Ersöz, Savaş Körlü, Derya Ümit Talas

**Affiliations:** 1 Yedikule Chest Disease Hospital, Istanbul, Turkey; 2 Mersin University Faculty of Medicine, Mersin, Turkey; 3 Mersin University Faculty of Medicine, Pediatric Surgery, Mersin, Turkey; 4 Mersin University Faculty of Medicine, Biophysic Department, Mersin, Turkey; 5 Mersin University Faculty of Medicine, Chest Disease Department, Mersin, Turkey; 6 Mersin University Faculty of Medicine, Pathology Department, Mersin, Turkey; 7 Mersin University Faculty of Medicine, Infectious Disease Department, Mersin, Turkey; 8 Mersin University Faculty of Medicine, Ear Nose Throat Department, Mersin, Turkey

**Keywords:** bevacizumab, trapidil, tracheal transaction, wound healing

## Abstract

**Background:**

Although bevacizumab has deleterious effects on the healing of colonic anastomoses, trapidil improves wound healing of colonic and tracheal anastomoses.

**Objective:**

We aimed to assess the effects of bevacizumab and trapidil on wound healing after tracheal transection.

**Materials and methods:**

We evaluated 35 rats divided in 5 groups: bevacizumab (Group I, n = 7), trapidil (Group II, n = 7), trapidil + bevacizumab (Group III, n = 7), controls (Group IV, n = 7), and sham (Group V, n = 7). Anastomotic healing was assessed by measurement of bursting pressure and inflammation score at the anastomotic region on the seventh day.

**Results:**

The bursting pressures of Group II, Group III, and Group V were significantly higher than controls (*P* = 0.001, *P* = 0.033, and *P* = 0.035, respectively). Fibrosis was significantly high in the sham group when compared with the other four groups (*P* = 0.047).

**Conclusions:**

Although bevacizumab seems to impair anastomotic healing, trapidil can be suggested to improve tracheal anastomoses.

## Introduction

Bevacizumab has become the standard therapy in a variety of solid tumors, including colorectal, kidney, gliablastome multiforme, and lung cancer.^1–3^ Many studies have demonstrated deleterious effects of agent targeting vascular endothelial growth factor on the healing of colonic anastomoses. Vascular endothelial growth factor-targeted therapies inhibit nitric oxide synthetase. By this way the release of nitric oxide will decrease.^3^

Trapidil is an antiplatelet agent that acts in part as a phosphodiesterase inhibitor and as a competitive inhibitor of the platelet-derived growth factor receptor. Trapidil, with its vasodilator and nitric oxide-releasing effects, may have some potential to diminish tissue injury.^4^

In our study, we aimed to investigate the effects of bevacizumab and trapidil on wound healing after tracheal transection.

## Materials and Methods

The study was approved by the Ethics Committe of Mersin University (No. 2011/04). The study was carried out at the Laboratory Animals of Mersin University. The animals were maintained in accordance with guidelines of the Committe on Care and Use of Mersin University Medical Faculty Animal Research Laboratory.

Thirty-five Wistar albino rats weighting 250 to 300 g were divided into 5 groups: Group I, single-dose bevacizumab⁎ 5 mg/kg (−1) intraperitoneally (n = 7); Group II, 12 mg/kg (−1)/day trapidil† orally 1 week (n = 7); Group III, single-dose bevacizumab and 1 week trapidil (n = 7); Group IV, control (n = 7); and Group V, sham (n = 7). The animals underwent tracheal transection and primary anastomoses with 7/0 vicryl (Ethicon, Inc, Somerville, New Jersey). Animals were anesthetized with 50 mg/kg intramuscular ketamine hydrochloride.‡ Vertical neck incision was performed and the trachea was exposed with microsurgical dissection. After careful hemostasis, transection was done between the fifth and sixth tracheal rings with microscissors. Then, the rings were immediately anastomosed with 7/0 vicryl under an operating microscope. During the procedure, suction was performed, if needed, to avoid any aspiration of secretion and blood. The neck was sutured under sterile conditions.

Anastomotic healing was assessed by measurement of bursting pressure and inflammation score at the anostomotic region on the seventh day. After transection of the trachea, a silastic catheter was inserted into the proximal side of trachea. The distal end was fixed to a pressure transducer and saline was infused through the catheter using a syringe pump at a rate of 2 mL/min. The pressure was monitored with Biopac MP-100 Acquisition System (Biopak Systems Inc, Goleta, California). Peak pressures documented first before rupture were recorded as the anastomotic bursting pressures.

The anastomotic horizontally oriented specimens were fixed in 10% formalin solution and embedded in parafin. Hematoxylen-eosin stained 4 μm-thick sections were evaluated for each case and also specimens were stained using Masson’s trichrome for detecting fibrosis. Among items for anastomotic healing inflammation, fibrosis and epithelial regeneration were semiquatitatively scored in the intercarilaginous area. The inflammation, fibrosis, and epithelial integrity were evaluated according to the modified Hyun Sung et al^5^ numerical scale. Fibrosis was graded between 1 and 3, where Grade 1 = indicates no fibrosis, Grade 2 = moderate fibrosis, and Grade 3 = widespread fibrosis. The inflammation, including lymphocytes and neutrophils, were graded as Grade 1 = indicating no inflammatory cells, Grade 2 = mild, Grade 3 = moderate, and Grade 4 = severe inflamation. Epithelial integrity, defined as the mucosal lining of the epithelium above the inflamation as the percentage of the mucosal lining of the epithelium (0%–100%), was semiquantitatively evaluated. All specimens were histopathologically detected with a Nikon Eclipse 80i bright field microscope (Nikon Instruments Inc, Melville, New York).

Data were represented as mean (SEM). Mann-Whitney U test was used for comparison of bursting pressures. *P* values < 0.05 were considered statistically significant.

## Results

**Fig. 1:**
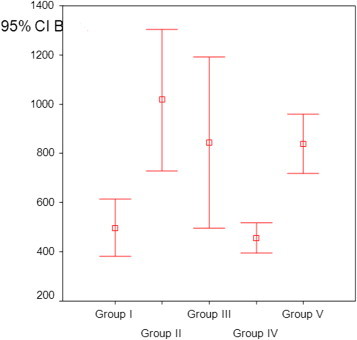
The bursting pressures of the groups. CI = confidence interval.

All animals survived the tracheal anastomoses procedure. No complication was observed. The bursting pressures of Group II, Group III, and Group V were significantly higher than the controls (*P* = 0.001, *P* = 0.033, and *P* = 0.035, respectively). The bursting pressure of Group I (bevacizumab group) was not different from the control group (*P* = 0.99). Because we are interested in wound healing the comparison should be made with the sham group instead of the control group. The bursting pressure of bevacizumab wass less than in the sham group and it could be assumed marginally significant (*P* = 0.073). Although the bursting pressure of sham group was significantly higher than controls (*P* = 0.035) there was no difference when compared with other groups (*P* = 0.62, Group II; *P* = 1.0, Group III) (Figure 1). The bursting pressure of the trapidil group was higher than that of the bevacizumab group (*P* = 0.002).

**Fig. 2:**
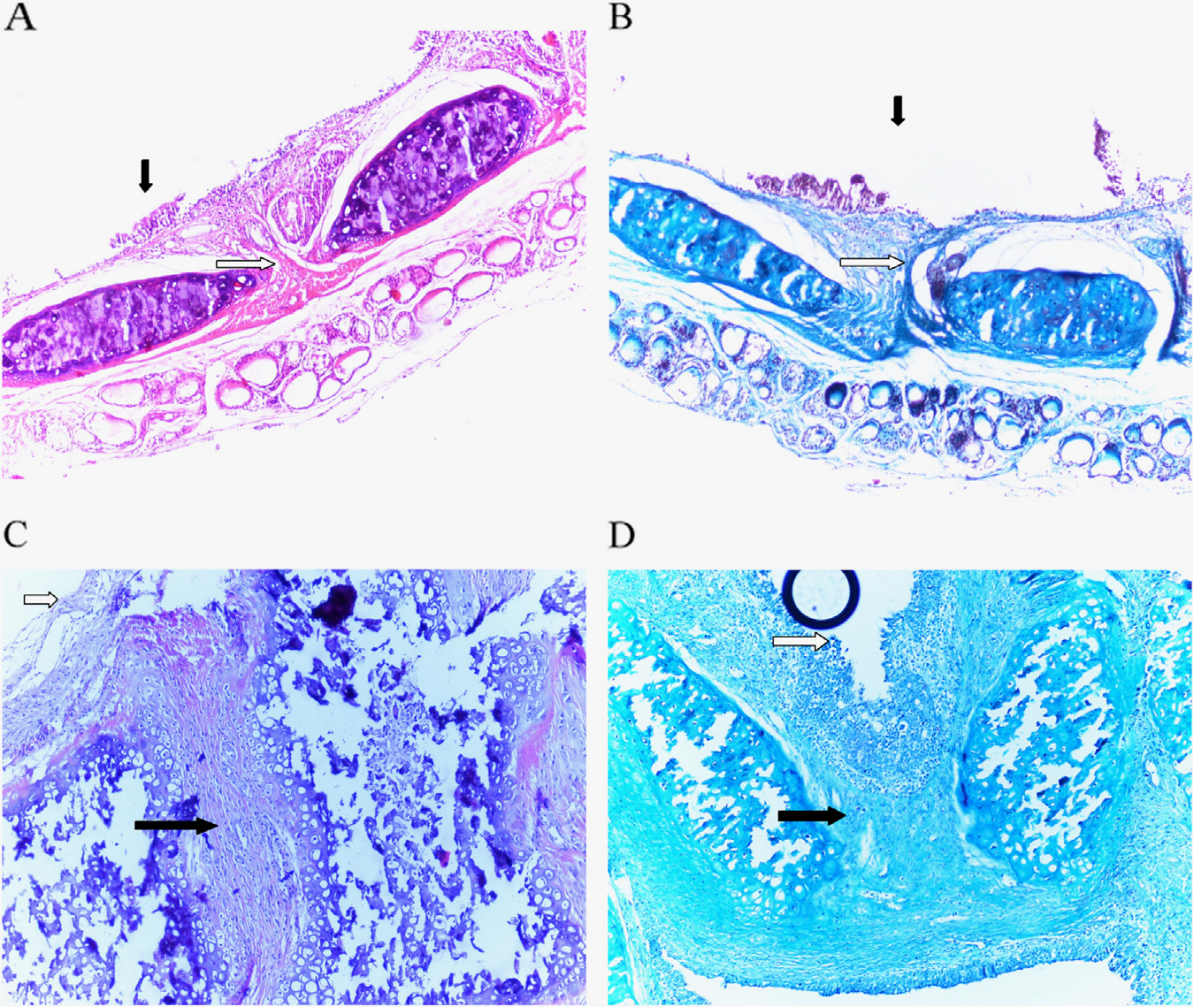
(A) Horizontal-section of tracheal anastomoses in a control rat (hematoxylin and eosin stain × 100). The histopathologic examination demonstrated the surface epithelium (black arrow), mild inflammation, and no fibrosis (white arrow) in the intercartilaginous area. (B) Horizontal-section of tracheal anastomoses in a control rat (trichrome stain × 100). No fibrosis is visible in the intercartilaginous area. (C) Horizontal-section of tracheal anastomoses in a sham rat (hematoxylin and eosin stain × 100). Histopathologic examination revealed slauthing epithelium (white arrow), moderate inflammation, and severe fibrosis (white arrow) in the intercartilaginous area. (D) Horizontal-section of tracheal anastomoses in a sham rat (trichrome stain × 100). Histopathologic examination revealed multilayered surface epithelium (white arrow) as swell as intense and widespread fibrosis (black arrow) in the intercartilaginous area.

There was a significant difference between Group I, Group II, Group III, and Group IV when compared with the sham group regarding the inflammation score for fibrosis (Figure 2). However, there were no differences between the groups with respect to neutrophils, lymphocytes infiltration, and epithelial integrity.

## Discussion

Our study revealed that bevacizumab, which impairs blood vessel growth, may interfere with wound repair and trapidil has a considerable healing effect on tracheal anastomoses. To the best of our knowledge, ours is the first study investigating bevacizumab in tracheal bursting pressure after tracheal transection in the English-language literature.

Although bevacizumab did not seem to impair anastomotic healing at the administered dose compared with the control animals, this is not the case if compared with the sham group. Intercartilaginous tissue is very thin and delicate in normal trachea. However, postoperative healing tissue formed at the end of the first week is thicker macroscopically and is histologically shown to have dense fibrous architecture. Therefore, it seems more logical to compare all the groups to sham-operated animals instead of the controls. Lower bursting pressure of the bevacizumab group compared with sham animals may be interpreted as impairment of wound healing. Although the bursting pressure of the sham group was higher than the bevacizumab group it did not reach statistical significance (*P* = 0.073). Trapidil increased the impaired bursting pressure of bevacizumab application. Histologic findings also contribute to this phenomenon.

Angiogenesis is a necessary step in wound healing. Nitric oxide is an important mediator in the angiogenesis. Bevacizumab can cause delayed or abnormal wound healing, wound dehiscence and hemorrhage. Bevacizumab inhibits nitric oxide synthase. Although we did not measure the nitric oxide level, it has been reported that inhibition of nitric oxide synthesis has deleterious effects on tracheal anastomotic healing.^6^ Bevacizumab in serum has a half-life of 20 days. Bronchogenic carcinoma involving the carina or tracheobronchial angle needs special surgical technique and airway management. Neoadjuvant combination of chemotherapy and bevacizumab is 1 of the strategies that can be used in these patients.^7,8^ We conclude that it is important to take into consideration the time of operation for wound healing among patients with bronchogenic carcinoma treated with bevacizumab. The upper airways and bronchial walls have similar histologies. Because it is difficult to make anastomoses in the bronchial tree of rats we investigated the effect of bevacizumab and trapidil on wound healing after tracheal transection.

Trapidil has various properties, including vasodilation, reduction of the inflammatory response to injury, inhibition of lipid peroxidation, and platelet aggregation. It has been reported that trapidil may improve wound healing in colonic and tracheal anastomoses in rats.^4,9^ These results are consistent with ours.

## Conclusions

Although bevacizumab seems to impair anastomotic healing, trapidil can be suggested to impove the tracheal anastomoses after the transection process. Further studies are needed to address the exact mechanisms of this result.

## Conflicts of Interest

The authors have indicated that they have no conflicts of interest regarding the content of this article.

## Footnotes

^⁎^ Trademark: Avastin^®^ (Roche, Basel, Switzerland).

^†^ Trademark: Rocarnal^®^ (Rentschler-UCBGmbH, Kerpen, Germany).

^‡^ Trademark: Ketalar^®^ (Eczacibasi-WL, Istanbul, Turkey).
